# The isoflavone metabolite 6-methoxyequol inhibits angiogenesis and suppresses tumor growth

**DOI:** 10.1186/1476-4598-11-35

**Published:** 2012-05-14

**Authors:** Sofia Bellou, Evdoxia Karali, Eleni Bagli, Nawaf Al-Maharik, Lucia Morbidelli, Marina Ziche, Herman Adlercreutz, Carol Murphy, Theodore Fotsis

**Affiliations:** 1 Department of Engineering Informatics and Telecommunications, University of Western Macedonia, 50100, Kozani, Greece; 2 Department of Biomedical Research, Foundation of Research and Technology-Hellas, Institute of Molecular Biology & Biotechnology, University Campus, 45110, Ioannina, Greece; 3 School of Chemistry, University of St Andrews, St Andrews, Fife, KY16 9ST, Scotland, UK; 4 Department of Molecular Biology, University of Siena, Via Aldo Moro 2, 53100, Siena, Italy; 5 Folkhälsan Research Centre, and Department of Clinical Chemistry, Biomedicum, University of Helsinki, P.O. Box 63, FIN-00014, Helsinki, Finland; 6 Laboratory of Biological Chemistry, Medical School, University of Ioannina, 45110, Ioannina, Greece

**Keywords:** Angiogenesis, VEGF, Phytoestrogen, MAPK, A-431 xenograft

## Abstract

**Background:**

Increased consumption of plant-based diets has been linked to the presence of certain phytochemicals, including polyphenols such as flavonoids. Several of these compounds exert their protective effect via inhibition of tumor angiogenesis. Identification of additional phytochemicals with potential antiangiogenic activity is important not only for understanding the mechanism of the preventive effect, but also for developing novel therapeutic interventions.

**Results:**

In an attempt to identify phytochemicals contributing to the well-documented preventive effect of plant-based diets on cancer incidence and mortality, we have screened a set of hitherto untested phytoestrogen metabolites concerning their anti-angiogenic effect, using endothelial cell proliferation as an end point. Here, we show that a novel phytoestrogen, 6-methoxyequol (6-ME), inhibited VEGF-induced proliferation of human umbilical vein endothelial cells (HUVE) cells, whereas VEGF-induced migration and survival of HUVE cells remained unaffected. In addition, 6-ME inhibited FGF-2-induced proliferation of bovine brain capillary endothelial (BBCE) cells. In line with its role in cell proliferation, 6-ME inhibited VEGF-induced phosphorylation of ERK1/2 MAPK, the key cascade responsible for VEGF-induced proliferation of endothelial cells. In this context, 6-ME inhibited in a dose dependent manner the phosphorylation of MEK1/2, the only known upstream activator of ERK1/2. 6-ME did not alter VEGF-induced phosphorylation of p38 MAPK or AKT, compatible with the lack of effect on VEGF-induced migration and survival of endothelial cells. Peri-tumor injection of 6-ME in A-431 xenograft tumors resulted in reduced tumor growth with suppressed neovasularization compared to vehicle controls (P < 0.01).

**Conclusions:**

6-ME inhibits VEGF- and FGF2-induced proliferation of ECs by targeting the phosphorylation of MEK1/2 and it downstream substrate ERK1/2, both key components of the mitogenic MAPK pathway. Injection of 6-ME in mouse A-431 xenograft tumors results to tumors with decreased neovascularization and reduced tumor volume suggesting that 6-ME may be developed to a novel anti-angiogenic agent in cancer treatment.

## Introduction

Physiological angiogenesis is a strictly regulated fine-tuned process. The local balance between inducers and inhibitors of angiogenesis is critical in determining the generation or not of new vessels. Whenever this balance is perturbed pathological, uncontrolled, excessive angiogenesis occurs. Psoriasis, rheumatic arthritis and diabetic retinopathy constitute some of the diseases in which pathological angiogenesis contributes to their pathogenesis. However, tumor angiogenesis is the most striking manifestation of abnormal angiogenesis. Indeed, it has been demonstrated that formation of new blood vessels is required for tumor growth beyond a diameter of 1-2 mm.

Vascular endothelial growth factor A (VEGFA), also referred to as VEGF, represents a critical inducer of tumor angiogenesis and is the first-choice target of anti-angiogenic therapies tested in clinical trials [1]. VEGF belongs to a subfamily of secreted, dimeric glycoproteins of approximately 40 kDa, which in turn belongs to the platelet-derived growth factor (PDGF) superfamily. In mammals, VEGF family consists of VEGF-A, B, C, D and placental growth factor 1 and 2 (PlGF1 and 2). Specifically VEGF exists as multiple isoforms, resulting from alternative splicing. The most predominant isoform is VEGF165 (a 165-amino acid protein), which is over-expressed in a variety of human solid tumors [2,3]. All VEGF molecules/ligands transduce their signal through their binding to VEGF receptor −1, -2 and −3. However, VEGFR-2 is the key molecule for VEGF signaling in the tumor micro-environment including vascular permeability and endothelial cell proliferation[2,4]. Several cascades emanating from the VEGF/VEGFR2 complex regulate critical angiogenic responses of endothelial cells. Endothelial cell proliferation is regulated by activation of PLCγ, a SH2-domain-containing molecule that interacts directly with activated VEGFR-2 and mediates the phosphorylation of mitogen-activated protein kinase (MAPK)/extracellular – signal-regulated kinase 1/2 (ERK1/2) cascade [5]. VEGF enhances survival of endothelial cells using the PI3K/AKT pathway, whereas it stimulates endothelial cell migration through p38 MAPK phosphorylation [6]. Signaling cascades of the VEGF/VEGFR2 complex result in the expression of dual specificity phosphatases (DUSP) 1 & 5, which dephosphorylate and inactivate MAPKs, functioning as an auto-regulatory circuit [7].

Consumption of plant-derived diets exerts a preventive effect on cancer incidence in humans. Several dietary phytochemicals exhibit anti-mitotic and/or anti-angiogenic activity mediating the protective effect of vegetarian diets on cancer. In this context, we have demonstrated that the isoflavonoid genistein is a potent inhibitor of tumor cell proliferation and angiogenesis [8]. Subsequently, we have shown that several of the isomeric flavonoids exhibited similar anti-angiogenic activity as genistein [9]. In particular, luteolin inhibited VEGF-induced angiogenesis by targeting VEGF/VEGFR2-induced PI3K activity. Detailed elucidation of the mechanism demonstrated that luteolin compromised VEGF-induced survival of HUVECs via blockage of PI3K/Akt-dependent pathways, whereas inhibition of the PI3K/p70 S6K pathway mediated the anti-mitotic effects of the compound on HUVECS [10]. In the present study, we have screened additional isoflavonoids for anti-angiogenic activity and identified that 6-methoxyequol inhibits VEGF-induced MEK1/2 phosphorylation and endothelial cell proliferation leaving unaffected the migratory and survival functions of VEGF. Treatment of xenograft A-431 tumors in mice using oral administration of 6-ME failed to reduce the volumes of the tumors, because the compound failed to achieve sufficient plasma levels as documented using an HPLC-CEAD method. However, injecting directly 6-ME to the xenograft tumors, to bypass the low bioavailability, resulting in a statistically significant reduction of tumor volume compared to controls and suppressed vascularization.

## Materials and methods

### Antibodies and chemicals

Human VEGF165 was purchased from ImmunoTools (ImmunoTools GmbH, Friesoythe, Germany). Rabbit polyclonal anti-phospho-p38, anti-ERK1/2, anti-phospho-ERK1/2, anti-phospho-Akt and anti-Akt antibodies were obtained from Cell Signaling (Cell Signaling Technology, Inc, Beverley, MA). Anti-BrdU was from Sigma (Sigma, St. Louis, MO). All secondary antibodies were purchased from Jackson ImmunoResearch Europe Ltd, UK. CycleTEST PLUS DNA Reagent kit was from Becton Dickinson Biosciences.

### Cell culture

Human endothelial cells from umbilical vein (HUVEC) were plated on dishes pre-coated with rat collagen type I (Becton Dickinson Biosciences) and cultured in M199 medium supplemented with 20% fetal calf serum (FCS), 50 micrograms/ml endothelial cell growth supplement (ECGS, Sigma), heparin 10u/μl (Sigma) and 1% penicillin-streptomycin. All media and sera for cell culture were purchased from Invitrogen and were endotoxin-free. 6-methoxyequol was tested for endotoxin content using the QCL1000 kit from BioWhittaker, Inc. For all experiments 6-methoxyequol was resuspended in DMSO/ethanol, 1/1 by volume, and added directly to the culture medium. Cells not receiving 6-methoxyequol were incubated in the corresponding volume of DMSO/ethanol.

### Primary cell growth assay

Primary bovine brain capillary endothelial (BBCE) cells were split into 12-well dishes at 5,000 cells per well and 24 h later cell stimulated with FGF2 (2.5 ng/ml) in the absence or presence of 6-methoxyequol at various concentrations. After 2 days, cells were again stimulated or not by FGF2 in the absence or presence of 6-methoxyequol and the next day cells were counted.

### Cancer cell growth assay

Hela, LnCAP, T24 (Human bladder carcinoma) or MCF7 (Human breast adenocarcinoma) cancer cells were split into 12-well plates either at 5,000, in case of Hela, T24 and MCF7 or at 20,000 in case of LnCAP, cells per well and 24 h later cells were treated or not with various concentrations of 6-methoxyequol. After 2 days, cells were again treated or not with 6- methoxyequol and the next day cells were counted.

### Apoptosis assay

For analysis by flow cytometry, HUVECs were serum starved for 6 h in medium containing 5% FCS and treated with VEGF (50 ng/ml) for 18 h in the presence or absence of 6-methoxyequol (10 μM) for the same period of time. At the end of the incubation time, floating and adherent cells were collected in ice-cold PBS, stained with propidium iodine using the CycleTEST PLUS DNA Reagent kit and processed for flow cytometric analysis using a Becton Dickinson Fluorescence Activated Cell Scanner (FACS). The percentage of cells with sub-G1 DNA content was considered as the cell population that had undergone apoptosis.

### Proliferation assay (BrdU incorporation)

HUVECs were grown on collagen-coated coverslips and serum starved in medium containing 5% FCS, 1% pen/strep and heparin for 18 h. Cells were induced with VEGF (50 ng/ml) in the absence or presence of various concentrations of 6-methoxyequol for 24 h. Bromodeoxyuridine (BrdU; Sigma, St. Louis, MO) was added 6 h before the VEGF-induction was complete. Cells were fixed in 3.7% paraformaldehyde, quenched with 50 mM ammonium chloride for 15 min, permeabilized with 0.1% Triton X-100 for 4 min, and non-specific sites were blocked with fetal serum. The proliferating cells were detected with an anti-BrdU antibody. Coverslips were mounted in Mowiol and viewed using Leica DM IBRE microscope.

### Cell migration assay

Confluent HUVE cell monolayers were wounded with a sterile plastic pipette tip, cultured in M199 medium supplemented with 5% FCS and induced with VEGF (10 ng/ml) in the presence or absence of 6-methoxyequol (10 μM). Cells were placed in a 37°C, 5% CO2 chamber and monitored using a Leica DM IBRE microscope equipped with a HRD060-NIK CCD-camera (Diagnostic Instruments, Sterling Heights, MI, USA) and metamorph software. Frames were taken every 10 min for 16 h. Results were expressed as number of cells per centimetre of wound.

### Tube formation assay

Matrigel was thawed on ice overnight and spread evenly over each well (500 μl) of a 24-well plate. The plates were incubated for 30 min at 37°C to allow the matrigel to polymerize. HUVECs were seeded on coated plated at 4 x 10^4^cells/well in M199 supplemented with 5% FCS in the presence or absence of 6-methoxyequol at various concentrations (1-50μM). Plates were incubated for 12 h at 37°C. Tube formation was observed using an inverted phase contrast microscope (**Zeiss** Axiovert **S-100**; Germany).

### Phosphorylation of MAP kinases

HUVECs were cultured in M199 supplemented with 20%FCS, ECGS, heparin & pen/strep until 80% confluence. Cells were serum starved for 2 h in medium containing 0% FCS and then treated with VEGF (50 ng/ml) in the presence or absence of either 6-methoxyequol (1,5,or 10μM) or DMSO for 15 min. Cells were washed with ice-cold PBS and lysed in lysis buffer (1% SDS supplemented with protease and phosphatase inhibitors). The lysates were resuspended in Laemmli buffer, subjected to SDS-PAGE and blotted onto a nitrocellulose membrane. Phosphorylated ERK1/2 and p38 were detected using specific rabbit polyclonal antibodies and an anti-rabbit peroxidase-conjugated secondary antibody, followed by detection using a chemiluminescence-based system. The membranes were then stripped and reprobed with antibodies against ERK1/2 and p38 to normalize the phosphorylation data against expression of the kinases.

### qRT-PCR experiment

Quantitative Reverse Transcription-PCR (qRT-PCR) experiments were performed using The LightCycler® 2.0 Instrument (Roche Diagnostics GmbH, Mannheim, Germany) and QuantiTect SYBR Green RT-PCR Kit (Qiagen, GmbH, Germany). Total RNA was isolated after 15 and 30 min treatment with VEGF (50 ng/ml) in the absence or presence of 6- methoxyequol (10μM).

### Synthesis of 6-methoxyequol

To test 6-ME in animal models considerably larger quantities were required. Since, this compound is not commercially available we undertook its synthesis as described in detail in the Additional file 1. In brief, starting from 6-methoxyresorcinol and 4-hydroxyphenylacetic acid the desired deoxybenzoin was first obtained in 48% yield. Treatment of the deoxybenzoin with *N*,*N*-dimethylformamid (DMF) in the presence of methanesulfonyl chloride at 70°C generated glycitein, which was hydrogenated using 10% Pd/C to 6-methoxyequol in high yield and purity. A detailed analysis of 1-(2,4-dihydroxy-5-methoxyphenyl)-2-(4’-hydroxyphenyl) ethanone, 7,4’-Dihydroxy-6-methoxyisoflavone (Glycitein) and 7,4’-Dihydroxy-6-methoxyisoflavane synthesis is described in.

### *In vivo* experiments

To assess the *in vivo* anti-angiogenic/anti-tumor activity of 6-methoxyequol, female immunodeficient mice (5–8 week-old BALB/c nude mice, Charles River, Milan, Italy), kept with ad libitum water and Protein Rodent Maintenance Diet (Harlan n. 2014), were inoculated subcutaneously in the right flank with 107 A-431 cells in a volume of 50 μl (Morbidelli et al., Clinic Cancer Res, 2003; Bagli et. al., Cancer Res, 2004). After 9 days, when tumors reached a volume of 170 mm^3^, animals were randomly assigned to 2 different experimental groups (9–10 mice per group). Peri-tumor treatment with 6-methoxyequol (5 μg/day/mice) or vehicle then began. The local peri-tumor treatment was performed at the dose of 5 μg/50 μl/mouse/day. The vehicle containing the same concentrations of solvents (1% ethanol + 1% DMSO) was used as control. Daily treatment was performed for 10 consecutive days. Serial caliper measurements of perpendicular diameters were used to calculate tumor volume using the following formula: (shortest diameter x longest diameter x thickness of the tumor in mm). Data are reported as tumor volume in mm^3^. Experiments have been performed in accordance with the guidelines of the European Economic Community for animal care and welfare (EEC Law No. 86/609) and National Ethical Committee. Animals were observed daily for signs of cytotoxicity and were sacrificed by CO_2_ asphyxiation. At day 10 animals were sacrificed and each tumor was immediately frozen in liquid nitrogen. 7 μm-thick cryostat sections were stained with hematoxylin and eosin and adjacent sections were used for immunohistochemical staining with the anti-ED-B monoclonal antibody after fixation in absolute cold acetone.

In the set of mice treated orally with 6-ME, the compound was firstly dissolved in 50% ethanol and 50% DMSO and then diluted with extra pure olive oil (final 0,25% ethanol and 0,25% DMSO). We have used as vehicle olive oil with the same amount of solvents. The daily dose of 6-ME was 100 mg/kg administered by lavage (200 μl/mouse). Treatment started when tumors were palpable and continued until day 11, the day of sacrifice. To accesses 6-ME bioavailability in mice, we determined 6-ME in urine and plasma as described in Additional file 1.

## Results

### Screening of flavonoids revealed that 6-methoxyequol is a specific inhibitor of endothelial cell proliferation exhibiting minor anti-mitotic effect on tumor cells

**Figure 1:**
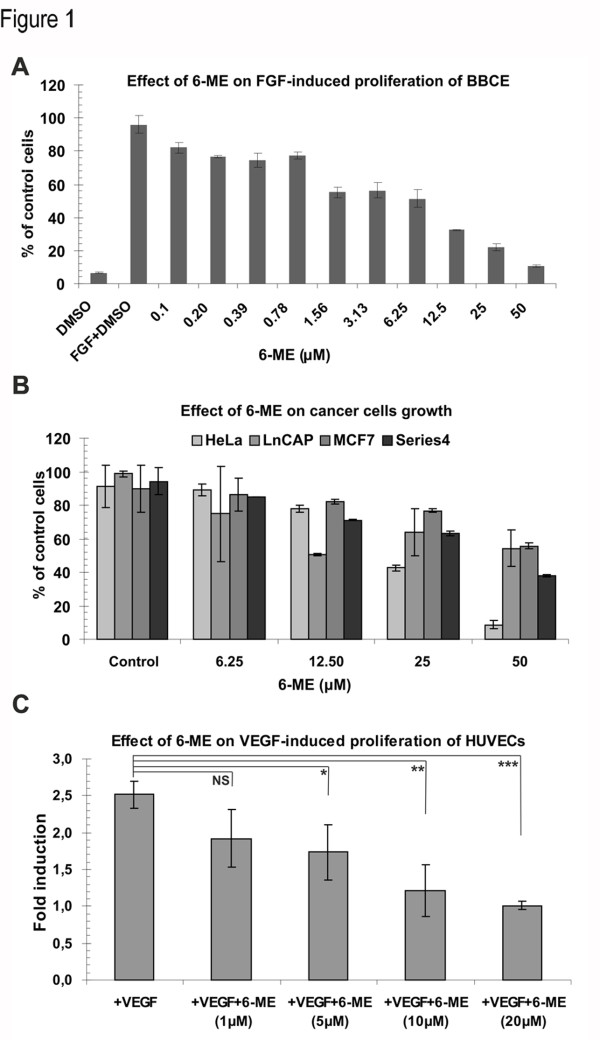
**Effect of 6-ME on endothelial and cancer cell proliferation. (A)** BBCE cells were seeded 24 h before stimulation by FGF (2.5 ng/ml) in the absence or presence of different concentrations of 6-ME. After 48 h cells were again stimulated with FGF (2.5 ng/ml) in the absence or presence of 6-ME and next day cells were counted. **(B)** HeLA, LnCAP, MCF7 or T24 cells were treated or not with various concentrations of 6-ME. After 48 h 6-ME was added again and 24 h later cells were counted. **(C)** HUVE cells were serum starved for 6 h in 5% FBS M199 supplemented with heparin and pen/strep. Then cells were stimulated by VEGF (50 ng/ml) for 18 h in the absence or presence of various concentrations of 6-ME. BrdU was added 6 h before the VEGF-induction was complete. Then indirect immunoflourescence was performed and the cells were viewed using Leica IBRE microscope. Graph indicates percentage of BrdU-incorporated cells ± s.d. derived from four independent experiments. ***p < 0.0001, **p = 0.0013, *p = 0.0056.

We screened a selection of isoflavonoids on endothelial cell proliferation seeking to identify additional structures with antiangiogenic activity compared to that of genistein. From the 28 isoflavonoids tested, only 6-methoxyequol (6-ME) had a strong inhibitory effect on FGF2-induced endothelial cell (BBCE) proliferation exhibiting an IC50 of approximately 3 μM ( 1 and Figure 1A), slightly lower than that of genistein and luteolin (IC50 around 5μM) [9,10]. The antimitotic effect of 6-methoxyequol appeared to be specific to endothelial cells as 6-ME was devoid of any antimitotic effect on 4 different cancer cell lines at a concentration of 6.25μM, even though at higher doses an inhibitory/toxic effect could be observed (Figure 1B). Moreover, 6-ME did not affect proliferation of human fibroblasts even at 20μM concentration (Additional file 1: Figure 1A and B). The inhibitory effect of 6-ME on endothelial cells was consistent as it inhibited also VEGF-induced proliferation of HUVECs (Figure 1C).

**Table 1:** IC50 list of isoflavonoids tested on endothelial cell proliferation

	**Compound**	**IC50(μM)**
**1**	3’,8-Dinitro-7-hydroxy-4-methoxyisoflavone	>50
**2**	7-Hydroxy-4-methoxy-3’,5’,8-trinitroisoflavone	45.9
**3**	4,7-Dihydroxy-3’,5’,8-trinitroisoflavone	>50
**4**	3’-Nitro-2,4,4’-trihydroxydeoxybenzoin	>50
**5**	2,4-Dihydroxy-4’-methoxy-5-nitrodeoxybenzion	>50
**6**	5,7-Dihydroxy-4’-nitroisoflavone	14.1
**7**	7-Hydroxy-4’-methoxy-8-nitroisoflavone	>50
**8**	3’-Nitro-5,7,4’-trihydroxyisoflavone	35.2
**9**	7,4’-Dihydroxy-3’-nitroisoflavone	42.3
**10**	5,7-Dihydroxy-2’-methyl-4’-nitroisoflavone	19.5
**11**	8,4’-Dinitro-7-hydroxyisoflavone	>50
**12**	4’-Amino-5,7-dihydroxyisoflavone	>50
**13**	8’-Amino-7-hydroxy-4’-methoxyisoflavone	31.7
**14**	3’-Amino-5,7,4’-trihydroxyisoflavone	24.8
**15**	3’-Amino-7,4’-dihydroxyisoflavone	37.5
**16**	4’-Amino-5,7-dihydroxy-2’-methylisoflavone	>50
**17**	4’,8-Diamino-7-hydroxyisoflavone	>50
**18**	8,4’-Diamino-7,5,dihydroxyisoflavone	>50
**19**	7,4’-Dihydroxy-8,5’-dinitroisoflavone	42.2
**20**	4’-methylequol	28
**21**	3’-methoxyequol	19
**22**	**6-methoxyequol**	**3**
**23**	6’-OH-ODMA	38
**24**	4’-O-methylequol	25
**25**	7’-Hydroxyenterolactone	>50
**26**	Pinoresinol	38
**27**	Luteolin	5
**28**	Genistein	5

### 6-methoxyequol does not influence VEGF-induced survival of endothelial cells

**Figure 2:**
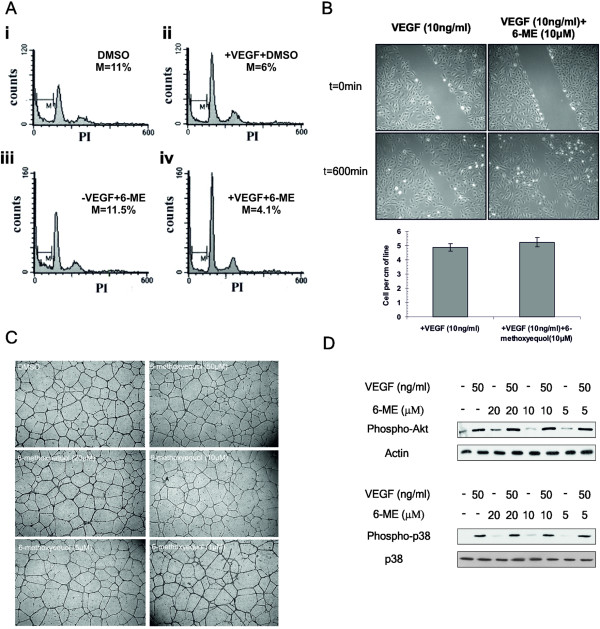
**Effect of 6-ME on VEGF-induced survival, migration of endothelial cells and tube formation *in vitro *and phosphorylation of Akt and p38 MAPK. (A)** HUVE cells were serum starved in 5% FBS M199 supplemented with heparin and pen/strep. Then cells were stimulated or not with VEGF (50 ng/ml) in the presence or absence of 6-ME (10μM) for 18 h. Floating and adherent cells were analyzed by flow cytometry. The number indicates the percentage of the hypodiploid cells. The experiment shown is a representative one from three independent experiments. **(B)** Confluent HUVE cell monolayers were wounded with a sterile tip prior to serum-starvation in M199 supplemented with 5% FBS, heparin and pen/strep. Then, cells were induced by VEGF (50 ng/ml) in the presence or absence of 6-ME (10μM) and placed in a 37°C, 5% CO_2_ chamber and monitored using a Leica DM IBRE microscope equipped with a HRD060-NIK CCD-camera and metamorph software. The graph shows images taken from non-induced cells at time points 0 and 600 min (at 600 min we have the possibility of contribution from proliferation – I would show an earlier time point) and from induced cells at the same time points, in the presence or absence of 6-ME, representing the number of the cells per centimeter of wound ± s.d. derived from three independent experiments. **(C)** HUVE cells were seeded on polymerized matrigel in M199 supplemented with 5% FBS, 1% pen/strep and heparin at density 8 x 10^4^ cell/ml. Then cells were treated with DMSO or 6-ME at various concentrations for 12 h and tube formation was observed using an inverted microscope. **(D)** HUVE cells were serum starved for 2 h in M199 and then stimulated with VEGF (50 ng/ml), in the absence or presence of 6-ME, for 15 min. Then cell lysates were collected with 1% SDS lysis buffer, supplemented with PMSF, and immunoblotting followed using antibodies against endogenous phospho-Akt, actin, phospho-p38 and p38. Experiments shown in (A) and (B) are representative from three independent experiments.

To exclude an inhibitory (apoptotic) effect of 6-ME on VEGF-induced survival of endothelial cells, we tested the effect of 6-ME on VEGF-treated endothelial cells following serum starvation. Withdrawal of serum is well known to induce endothelial cell apoptosis, which is reversible upon VEGF addition. Indeed, while 11% of the HUVECs were apoptotic, being hypodiploid in FACS analysis, after serum starvation (in 5% FCS) (Figure 2Ai), treatment with VEGF for 18 h rescued almost 50% (6% apoptotic cells) of the cells from apoptosis (Figure 2Aii). Upon treatment of serum deprived HUVECs with 10 μM concentration of 6-ME, 11.5% of HUVECs were apoptotic (Figure 2Aiii) showing no difference to the DMSO control (11% in Figure 2Ai). Finally, treatment of serum starved HUVECs with 10 μM of 6-ME did not affect the VEGF-induced survival of endothelial cells (4.1% apoptotic cells in Figure 2Aiv). The above observations were further confirmed using Annexin/PI-apoptosis assay (Additional file1: Figure S2). These results strongly suggested that 6-ME had no effect on the survival cascades of VEGF.

### 6-methoxyequol does not inhibit migration of endothelial cells and tube formation *in vitro*

Next, we investigated the possibility that 6-ME could inhibit other processes of angiogenesis. Indeed, angiogenesis is a complex process requiring the coordinated, sequential involvement of a number of cellular events. Formation of new capillaries begins with a localized breakdown of the basement membrane of the parent vessel, followed by migration of endothelial cells for invasion of the surrounding matrix. There, a cell-matrix mediated outgrowth of an endothelial tip cell is followed by stalk cell proliferation and eventually by tube formation with an encased lumen sealed by tight cell-cell junctions. The endothelial cell migration assay and the *in vitro* angiogenesis assay on Matrigel recapitulate reasonably well these early events of angiogenesis. 6-ME, at 10 μM concentration, did not influence the VEGF-induced migration of endothelial cells in wounded confluent monolayers of HUVECs (Figure 2B). Similarly, 6-ME, even at 50 μM concentration, did not perturb capillary-like tube formation of HUVECs plated on Matrigel (Figure 2C) or the structure of the cytoskeleton (Additional file1: Figure S3). Thus, 6-ME appears to affect only endothelial cell proliferation leaving unaffected other angiogenic responses of endothelial cells.

### 6-methoxyequol inhibits activation of the MEK1/2-ERK1/2 pathway by VEGF

**Figure 3:**
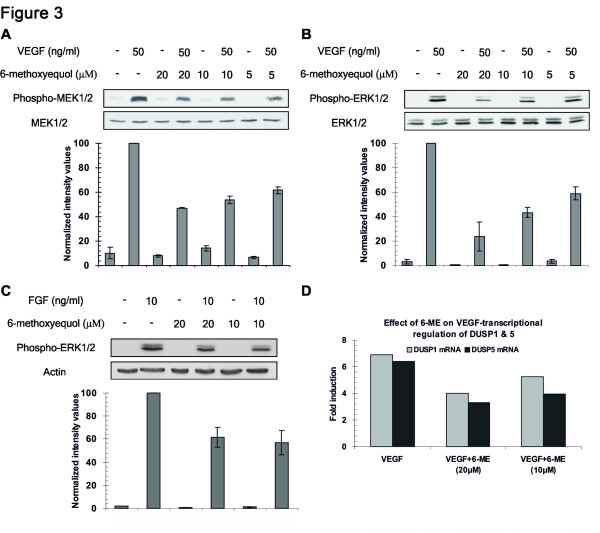
**Effect of 6-ME on VEGF-induced phosphorylation of MEK1/2 and ERK1/2 and transcription of DUSP1 and DUSP5.** HUVE cells were serum starved for 2 h in M199 and then stimulated with VEGF (50 ng/ml) **(A & B)** or FGF (2.5 ng/ml) **(C)**, in the absence or presence of 6-ME, for 15 min. Then cell lysates were collected with 1% SDS lysis buffer supplemented with PMSF and immunoblotting followed using antibodies against endogenous phospho-MEK1/2, MEK1/2, phospho-ERK1/2, ERK1/2 and actin. Graphs show normalized intensity values ± s.d. derived from three independent experiments. **(D)** HUVE cells were stimulated by VEGF (50 ng/ml) in the absence or presence of 6-ME (20, 10μM) for 30 min. Then, total RNA was isolated and qRT-PCR experiments followed using primers for *DUSP1* and *DUSP5.*

**Figure 4:**
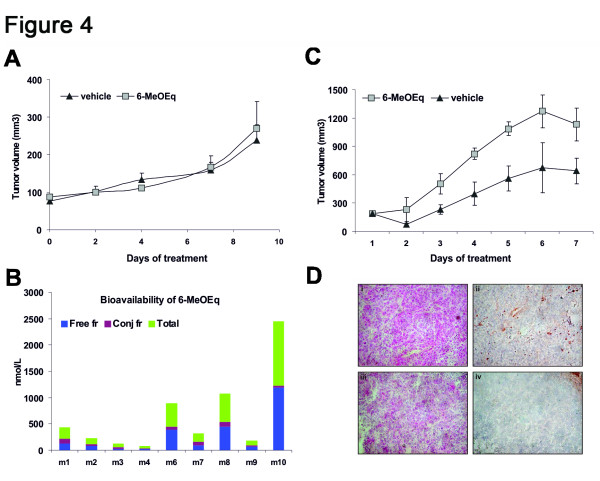
**In vivo experiments.** Female immunodeficient mice were inoculated subcutaneously in the right flank with 10^7^ A-431 cells in a volume of 50 μl. When tumors reached a volume of 100 mm^3^**(A & B)** or 170 mm^3^**(C & D)**, animals were randomly assigned to 2 different experimental groups and oral or peri-tumor treatment with 6-ME (5 μg/day/mice) or vehicle began, according to Materials and Methods. (A) Graph shows the tumor volume (mm^3^) ± s.d. derived from 9 animals. **(B)** Free fraction (Free fr), conjugated fraction (Conj fr) and total amount of 6-ME in the plasma of 9 animals (m1-m9) **(C)** Graph shows the tumor volume (mm^3^) ± s.d. derived from 9 animals in the case of 6-ME treated mice and 4 animals treated with vehicle, since the increased tumor volume and ulceration started from day 6 caused the death of 5 animals in this group. **(D)** The effect of 5 ug/day 6-ME (panels iii-iv) on tumor angiogenesis at day 10 was compared to vehicle treated group (panels i-ii). Representative pictures of tumor sections stained with hematoxylin and eosin (i,iii) and with the antibody specific for B-FN (ii,iv).

Having established that 6-ME inhibits only endothelial cell proliferation without affecting survival, migration and tube formation, we sought mechanistic confirmation of these findings. Indeed, 6-ME did not affect VEGF-induced phosphorylation of AKT (Figure 2D, upper panel), one of the key cascades that confer endothelial cell survival [11]. Likewise, 6-ME did not affect VEGF-induced phosphorylation of p38 MAPK (Figure 2D, lower panel), a signaling cascade that mediates the induction of endothelial cell migration by VEGF [12]. These results, together with the fact that 6-ME does not inhibit PLC-γ activation, as VEGF-induced calcium release in not affected (Additional file1: Figure 4), exclude the kinase activity of VEGFR2/KDR of being the target of 6-ME**.** In confirmation, 6-ME clearly inhibited, at 10μM concentration, the phosphorylation of MEK1/2 (Figure 3A) and its downstream target ERK1/2 (Figure 3B), components of the mitotic MAPK pathway that VEGF triggers via PLC-γ activation. Several growth factors activate the ERK1/2 MAPK pathway in a Ras-dependent manner [13,14]. Indeed, 6-ME inhibited also FGF2-induced phosphorylation of ERK1/2 fully compatible with the fact that 6-ME inhibited also FGF2-induced proliferation of BBCE cells (Figure 1A). To fully confirm inhibition of the ERK1/2 cascade by 6-ME, we sought additional evidence by investigating the transcriptional activation of DUSP1 and DUSP5 genes that are regulated by VEGF via the ERK1/2 pathway [15-18]. DUSP1 and DUSP5 are dual-specificity phosphatases that dephosphorylate ERK1/2 and p38 MAPK, being part of an auto-regulatory circuit [7]. Indeed, 6-ME clearly inhibited the induction of DUSP1 and DUSP5 mRNA levels by VEGF (Figure 3D) leaving no doubt that it inhibits VEGF-induced ERK1/2 activation.

### 6-methoxyequol inhibits xenograft tumor growth only when administered directly to the tumors

Next, we undertook the task of testing the compound in mouse xenograft tumor models. For this purpose, the synthesis of sufficient quantities of 6-ME was assured using acylation of 4-methoxyresorcinol with 4-hydroxyphenylacetic, followed by treatment of the resulting deoxybenzoin with *N*,*N*-dimethylformamid to yield glycitein, which was hydrogenated to 6-methoxyequol in high yield and purity (Additional file 1).

We used a murine tumor xenograft model utilizing A-431 cells, a human epidermoid carcinoma cell line that produces VEGF [19]. Since in previous studies we and others have found that isoflavonoids may exhibit low bioavailability [20,21], we decided to carry out two sets of experiments. In one set, 6-ME was administered orally in olive oil suspension whereas in the other, the compound was injected directly in the vicinity of the xenograft tumor.

6-ME administered orally in this model was devoid of any effect. The experimental and control tumors did not show any difference in their average volumes (Figure 4A) even though some of the experimental tumors were clearly smaller in volume compared to the control tumors (data not shown). We postulated that low bioavailability is the reason for the lack of effect. Indeed, estimation of the free, conjugated and total amounts of 6-ME in the plasma of the mice revealed that the maximum concentration achieved was 1.23 μM (Figure 4B), a value below the *in vitro* IC50 of the compound (around 5-10μM).

Injecting directly the A-431 tumors with 6-ME (5 μg/day/mice) reduced the growth of tumors compared to the control group treated with vehicle. Tumors in 6-methoxyequol treated mice were significantly smaller (approximately 50%, P < 0.01 *vs.* vehicle group at day 6 and 8) than in control mice beginning from day 2 (Figure 4C). B-fibronectin (B-FN), the fibronectin (FN) isoform containing extradomain B (ED-B) accumulates around neovascular structures in aggressive tumors and other tissues undergoing angiogenesis and remodeling [22]. The monoclonal anti-ED-B antibody against the ED-B domain in fibronectin [23] indicated the presence of tumor vasculature in tumors of the control group, which was absent in 6-methoxyequol treated tumors (Figure 4D). Regarding the survival, at day 8 mice survival was 78% in the 6- methoxyequol group and 44% in the vehicle group.

## Discussion

In previous studies, we have demonstrated that the isoflavonoid genistein is an angiogenesis inhibitor [8]. In the present study, we have screened a number of hitherto untested isoflavonoids using inhibition of EC proliferation as an indicator of possible anti-angiogenic activity. Only, 6-ME inhibited EC proliferation with an IC50 comparable to that of genistein or the flavonoid Luteolin (around 5 μM). Interestingly, 6-ME inhibited both VEGF- and FGF2-induced proliferation of endothelial cells, whereas it had no effect on the serum-induced proliferation of four cancer cell lines. Apparently, 6-ME exhibits certain selectivity towards inhibition of EC proliferation. 6-ME is an isoflavan metabolite that has been identified in human urine following soy or red clover supplementation [20,21,24,25]. However, only trace amounts of 6-ME are excreted in human urine. 6-ME originates from glycitein; the amount of the original substance is low in soy compared to daidzein and genistein, that may explain the low amounts of the metabolite [24].

Though 6-ME inhibited both VEGF- and FGF2-induced proliferation of ECs, we decided to study the effects of 6-ME only on VEGF-dependent EC responses, because VEGF is the most important mediator of tumor angiogenesis. Indeed, cancer cells over-express VEGF either following hypoxia or as a consequence of the genetic changes of cancer such as mutations of oncogenes and tumor suppressor genes [26]. In fact, endothelial cells adjacent to the tumor vessels over-express VEGFR-1 and −2 [27] establishing an angiogenic loop.

To discriminate whether the decreased number of cells in the proliferation assay derived from a truly cytostatic effect (cell cycle inhibition) of 6-ME or was the result of cytotoxicity/apoptosis, we further investigated the effect of the compound on the VEGF-induced survival of endothelial cells. 6-ME, administered alone to endothelial cell cultures did not increase the percentage of apoptotic cells compared to solvent-treated cultures. Moreover, 6-ME administered together with VEGF did not have any influence on the VEGF-induced rescue of apoptosis. This result, in other words, indicated that 6-ME did not inhibit the EC survival signaling cascades emanating from the active VEGF/VEGFR2 complex. In confirmation, 6-ME did not inhibit VEGF-induced phosphorylation of AKT, an important component of the PI3K signaling pathway, the main anti-apoptotic cascade in most cells.

Having established that 6-ME inhibits endothelial cell proliferation, we investigated whether 6-ME could inhibit other angiogenic responses of endothelial cells. Indeed, angiogenesis is a complex process that involves many partial steps such as production of proteolytic enzymes that degrade the basement membrane, migration, proliferation, tube formation, generation of basement membrane and recruitment of mural cells [26]. Several of these processes including tube formation can be reconstituted *in vitro* using 3D cultures on Matrigel, a basement membrane matrix from Engelbreth-Holm-Swarm mouse tumors [28]. Indeed, human umbilical vein endothelial cells form capillary-like structures on Matrigel substrates. 6-ME, even at high doses, did not exhibit any effect on the Matrigel assay. Migration is a critical angiogenic response of ECs allowing them to reach the membrane breach for invasion to the extracellular space. VEGF is a prime regulator of EC migration. VEGF-induced phosphorylation of Tyr1214 of VEGFR2 activates SAPK2/p38 [12] leading to VEGF-induced actin reorganization and migration of ECs via phosphorylation of heat-shock protein-27 (HSP27) [29] and LIM-kinase 1 (LIMK1) [6]. 6-ME did not exhibit any inhibitory effect on VEGF-induced migration of ECs and did not inhibit phosphorylation of p38 by the VEGF/VEGFR2 complex.

It appeared, therefore, that the main target of 6-ME was EC proliferation. Interestingly, 6-ME inhibited both VEGF- and FGF2-induced EC proliferation. In humans, upon VEGF-A binding, phosphorylation of VEGFR2 on Tyr1175 leads to recruitment of PLCγ, which in turn, via activation of PKC, phosphorylates MEK1/2 and eventually mitogen-activated protein kinase (MAPK)/extracellular-signal-regulated kinase-1/2 (ERK1/2) leading to proliferation of ECs [5]. Such activation of MAPKs by VEGF is different from classic Ras-Raf-MEK-MAPK pathway, which is used by most receptor tyrosine kinases including FGF2 [13,14]. Nevertheless, it has been shown that PKC-dependent activation of MEK1/2 requires a Ras-Raf complex formation [30]. This PKC/Ras-Raf functional interaction is not so well understood and might include other hitherto unidentified components. PKC and Ras-Raf are the points where the VEGF and FGF2 cascades arrive just before the first downstream common effector, MEK1/2, as far as activation of MAPK is concerned. The finding that 6-ME inhibits both the VEGF and FGF2-induced EC proliferation as well as MEK1/2 phosphorylation suggests that the PKC/Ras-Raf interaction is the only point where 6-ME could target both pathways with one activity. Otherwise, 6-ME would need two activities targeting two different components upstream to MEK1/2, one for each pathway. This is a point that requires future attention.

Thus, inhibition of MEK1/2 and consequently ERK1/2 phophorylation was the sole cardinal effect of 6-ME on the signaling cascade of VEGF in HUVECs; activation of AKT and P38 were unaffected. This mechanism is strikingly different compared to the effects of the flavonoid luteolin on VEGF signaling in HUVECs [10]. Luteolin, inhibited the PI3K/AKT pathway abolishing downstream survival signals, but also enhanced the pro-apoptotic MKK3/MKK6/p38 pathway of VEGF eliciting a strong apoptotic effect in ECs. Regarding the anti-mitotic activity, luteolin inhibited VEGF-induced phosphorylation of p70 S6K, a downstream effector of PI3K responsible for G1 progression. Surprisingly, luteolin did not affect VEGF-induced phosphorylation of ERK1/2 MAP kinases. Thus, two representatives (luteolin and 6-ME) of closely related isomeric compound classes (flavonoids and Isoflavonoids) exhibited entirely different molecular targets concerning the VEGF-dependent signaling cascades in HUVECs. Perhaps, the fact that these compounds are competitive inhibitors of ATP binding [31] allows them to target a variety of tyrosine and serine kinases [31,32].

6-ME was eventually tested in animal models. For this purpose, we used a murine tumor xenograft model utilizing A-431 cells, a human epidermoid carcinoma cell line that produces VEGF [19]. 6-ME administered orally in this model was devoid of any effect. The experimental and control tumors did not show any difference in their average volumes). We postulated that low bioavailability is the reason for the lack of effect. Indeed, estimation of the free, conjugated and total amounts of 6-ME in the plasma of the mice revealed that the maximum concentration achieved was 1.23 μM, a value below the *in vitro* IC50 of the compound (around 5-10μM). Several factors contribute to the bioavailability including absorption, distribution, metabolism and elimination. There are no extensive studies on these issues concerning isoflavonoids. However, the studies so far [20,21] anticipate that isoflavones are rather poorly bioavailable. In a study in human adults, consumption of 50 mg of isoflavones per day yielded plasma concentrations ranging from 0.2-3.2 μmol/L. Indeed, following consumption of food rich in soy or red clover only traces of 6-ME were detected in soy human urine [24]. The low biovailability excludes any significant contribution of 6-ME to the protective function of plant-based diets on cancer incidence.

However, biovailable analogs of 6-ME could be used therapeutically to target tumor angiogenesis. Alternatively, 6-ME could be loaded in nanoparticles targeted to ECs, where they could be endocytosed and eventually release their cargo. Indeed, when injected directly to the xenograft tumors, to bypass its low biovailability, 6-ME suppressed tumor vascularization resulting to a statistically significant decrease in the volumes of murine A-431 xenograft tumors. Thus, 6-ME acquires the potential to be developed into a therapeutic anti-cancer agent. In this capacity, 6-ME or 6-ME analogs have two very important and unique properties. 6-ME inhibits only VEGF-induced MEK1/2 activation inhibiting exclusively EC proliferation without influencing VEGF-induced survival. Thus, one can anticipate that it targets only dividing ECs in the vicinity of tumors, without affecting the survival of the quiescent normal endothelium. Moreover, it inhibits also FGF2, which an alternative angiogenic factor expressed when ECs develop resistance (Angiogenic Redundancy) [33] against current anti-VEGF treatments [34]. This is a very important issue in the anti-VEGF treatments.

In conclusion, 6-ME, a natural isoflavone found also in humans, inhibits VEGF- and FGF2-induced proliferation of ECs. The molecular target of 6-ME is upstream of MEK1/2 inhibiting phosphorylation of MEK1/2 and ERK1/2 kinases that are important components of the mitogenic MAPK pathway. 6-ME does not affect the PI3K/AKt pathway, thereby not affecting VEGF-dependent survival of ECs. Oral administration in mice fails to achieve sufficient plasma concentrations to inhibit neovascularization and growth of xenograft tumors in mice. However, direct injections of 6-ME to the xenograft tumors, to bypass its low biovailability, suppress tumor vascularization resulting to a statistically significant decrease in the volumes of murine A-431 xenograft tumors. Concomitant inhibition of VEGF- and FGF2-induced EC proliferation and targeting only dividing ECs without affecting the survival of ECs are two properties rendering 6-ME as an attractive molecule for the development of a novel anti-angiogenic intervention in cancer treatment.

## Competing interests

The authors declare that they have no competing interests.

## Supplementary Material

Additional file 1 Scheme 1: Synthesis of 6-Methoxyequol. **Additional file 1: Figure1** Effect of 6-ME on primary human fibroblast proliferation. **Additional file 1: Figure2** Effect of 6-ME on VEGF-induced survival of endothelial cells. **Additional file 1: Figure 3** Effect of 6-ME on the structure of actin filaments and microtubules. Click here for file
