# Supplementary material for: The isoflavone metabolite 6-methoxyequol inhibits angiogenesis and suppresses tumor growth
Source: Mol Cancer. 2012 May 14;11:35. doi: 10.1186/1476-4598-11-35 (PMC3406996; doi:10.1186/1476-4598-11-35)
Supplement: Additional file 1 — Scheme 1: Synthesis of 6-Methoxyequol. Additional file 1: Figure1 Effect of 6-ME on primary human fibroblast proliferation. Additional file 1: Figure2 Effect of 6-ME on VEGF-induced survival of endothelial cells. Additional file 1: Figure 3 Effect of 6-ME on the structure of actin filaments and microtubules. [file 1476-4598-11-35-S1.doc]

**SUPPLEMENTARY DATA**

**Scheme 1: Synthesis of 6-Methoxyequol**

6-Methoxyequol **5** was synthesized in two steps starting form 6-methoxyresorcinl **1** and 4-hydroxyphenylacetic acid **2** as outlined in Scheme 1. BF_3_.Et_2_O-mediated Friedel-Crafts acylation of 4-methoxyresorcinol **1** with 4-hydroxyphenylacetic acid **2** at reflux temperature gave the desired deoxybenzoin **3** in 48% yield. Treatment of **3** with *N*,*N*-dimethylformamid (DMF) in presence of methanesulfonyl chloride at 70 ^o^C afforded glycitein **4**, which on hydrogenation using 10% Pd/C gave 6-methoxyequol **5** in high yield and purity.

Synthesis of 1-(2,4-dihydroxy-5-methoxyphenyl)-2-(4’-hydroxyphenyl) ethanone (3): A mixture of 4-methoxyresorcinol **(1)** (1.3 g, 9.29 mmol) and 4-hydroxyphenylacetic acid (**2)** (1.27 g, 8.36 mmol) in BF_3_.Et_2_O (6.85 mL, 55.7 mmol) was refluxed for 15 min under nitrogen. After cooling to room temperature, the dark red solution was poured into ice-cold aqueous sodium acetate (100 mL) and extracted with ethyl acetate (50 mL x 3). The combined ethyl acetate layer was washed with 10% aq. NaHCO_3_ (50 mL) and water (100 mL), respectively, and dried over MgSO_4_. Solvent was evaporated under reduced pressure, and the dark red oil was subjected to chromatography (CH_2_Cl_2_:EtOAc = 8:2) to give the title compound (**3)** as a yellow solid (1.1 g, 48%), m.p. 157-159°C; *δ_H_* (300 MHz, DMSO-*d_6_*) 3.76 (s, 3H, OCH_3_), 4.16 (s, 2H, CH_2_), 6.30 (s, 1H, H-3), 6.69 (d, *J* = 8.6 Hz, 2H, H-3’,5'), 7.08 (d, *J* = 8.6 Hz, 2H, H-2’,6'), 7.40 (s, 1H, H-6), 9.29 (br s, 1H, OH), 10.48 (br s, 1H, OH), 12.38 (br s, 1H, OH). δ_C_ (75 MHz, DMSO-*d_6_*) 43.6 (C-CH_2_), 56.3 (C-OCH_3_), 103.2 (C-2), 110.3 (C-1), 113.3 (C-6), 115.6 (C-3',5'), 125.3 (C-1’), 130.4 (C-2',6’), 141.0 (C-5), 155.4 (C-2), 156.0 (C-4'), 159.3 (C-4), 202.2 (C-CO); *m/z* (EI) 315 (M^+^, 100%), 316 (18), 269 (6), 241 (7), 152 (17), 136 (3), 124 (5)MS (EI, 70 ev): m/z = 275 (M+ 1, 100%), 274 (16%), 257 (9%), 167 (18%); HRMS calcd for (C_15_H_14_O_5_) 274.9841, found 274.9829.

Synthesis of 7,4’-Dihydroxy-6-methoxyisoflavone (Glycitein) (4): BF_3_.Et_2_O (1.6 mL, 13.13 mmol) was added to a solution of 5-methoxy-2,4,4’-trihydroxydeoxybenzoin **(3)** (0.6 g, 2.19 mmol) in dry DMF (10 mL) under nitrogen at ambient temperature. After 15 min stirring, a solution of methanesulfunyl chloride (0.84 mL, 10.95 mmol) in dry DMF (2 mL) was slowly added and the solution was heated at 70 °C for 5 hrs. The reaction mixture was cooled down to ambient temperature and poured into ice-cold aqueous sodium acetate (50 mL), the yellow precipitate was filtered off and recrystallised from 70% ethanol to give **(4)** as a yellow solid (0.85 g, 85%), m.p. 336-338 °C( Lit.^2^ 337 ^o^C); *δ_H_* (300 MHz, DMSO-*d_6_*) 3.86 (s, 3H, OCH_3_), 6.79 (d, *J* = 8.7 Hz, 2H, H-3’,5’), 6.92 (s, 1H, H-8), 7.37 (d, *J* = 8.7 Hz, 2H, H-2’,6'), 7.41 (s, 1H, H-5), 8.27 (s, 1H, H-2), 9.54 (brs, 1H, 4’-OH), 10.63 (brs, 1H, 7-OH). MS (EI, 70 ev): m/z = 284 (M^+^, 24%), 283 (100%), 268 (15%), 255 (20%) 212 (41%), 171 (15%). HRMS calcd for (C_16_H_12_O_5_) 284.0685, found 284.0679.

Synthesis of 7,4’-Dihydroxy-6-methoxyisoflavane: Glycitein (0.5 g, 1.76 mmol) was reduced with H_2_ over 10% Pd/C (0.25 g) in ethanol (50 mL) until no more hydrogen was consumed. Pd/C was filtered off and solvent was evaporated. The residue was purified by chromatography over silica gel (CH_2_Cl_2_:EtOAc = 8:2) to give the title compound as a white solid (0.43 g, 90%) (from benzene), m.p. 159 °C; δ_H_ (300 MHz, CDCl_3_) 2.90-2.94 (m, 2H, H-4a,b), 3.11-3.24 (m, 1H, H-3b), 3.83 (s, 3H, OMe), 3.92 (t, *J* = 10.5 Hz, 1H, H-2a), 4.25 (ddd, *J* = 1.8, 3.6, 10.5 Hz, 1H, H-2b), 4.85 (s, 1H, 4’-OH), 5.54 (s, 1H, 7-OH), 6.48 (s, 1H, H-8), 6.55 (s, 1H, H-5), 6.81 (d, *J* = 8.9 Hz, 2H, H-3’,5'), 7.11 (d, *J* = 8.9 Hz, 2H, H-2’,6'). δ_C_ (75 MHz, DMSO-*d_6_*) 32.2 (C-4), 38.0 (C-3), 56.5 (C-5), 70.9 (C-2), 103.1 (C-8), 111.5 (C-5), 112.4 (C-4a), 115.6 (C-3',5'), 128.5 (C-2',6’), 133.8 (C-1’), 140.9 (C-6), 144.8 (C-7), 148.4 (C-8a), 154.5 (C-4’). *m/z* (EI) MS (EI, 70 ev): m/z = 273 (M + 1, 71%), 272 (100%), 258 (48%), 153 (18%); HRMS calcd for (C_16_H_16_O_4_) 272.1049, found 272.1059.

**Determination of 6-ME in mouse urine**

For the analysis, 100μl of urine were used. The free fraction was extracted with 2 x 1ml of diethylether (Merck). After extraction the sample water phases were hydrolyzed over night at 37°C by adding equal volume (100µl) of hydrolysis solution (acetate buffer pH5, 0.1M, β-glucuronidase 0.2U/ml (Roche Diagnostics) and sulphatase 2U/ml (Sigma)). After hydrolysis, the conjugated fraction was extracted with 2 x 2 ml of diethylether. The organic fractions were evaporated to dryness. Deuterated d6-6MeEquol in methanol was added to the samples, evaporated and the dry samples were silylated in 100µl of silylation reagent (pyridine:hexamethydisilazane:trimethychlorsilane 9:3:1 vol:vol:vol) for 30 min at room temperature and 1µl was injected and run in GCMS. The following ions were monitored: 192, 416 for 6-ME, 194, 419 for 13C-6-ME and 196, 422 for d6-6-ME

GCMS analysis was carried out on a BP-5 column using the following temperature program: 150°C/1min, increased 50°C/min ->220°C /0min increased 1.5°C/min -> 250°C/0min increased 50°C/min ->290°C/2min, total run time was 25.2 minutes. The Injector, interphase and source temperatures were 280°C, 270°C and 250°C respectively.

**Determination of 6-ME in mouse plasma**

For the analysis, the total amount of sample that was available was used, i.e. from 63-180µl of plasma. The free fraction extracted with 3 x 1ml of diethylether. After extraction the sample water phases were hydrolyzed similarly to the urine samples and after incubation the CONJUGATED fraction was extracted with 3 x 2ml of diethylether.

For HPLC-CEAD the samples were evaporated to dryness and re-dissolved in 100µl of methanol from which 10µl was injected to HPLC-CEAD. The analytical column was an Inertsil ODS-3 (GL Sciences Inc., Japan) 3 x 150 mm with particle size 3 µm, connected to a Quick Release RP-18 (Upchurch Scientific Inc., WA, USA) 3 x 10 mm guard column.

The analytes were separated using mobile phases consisting of 50 mmol/L acetate buffer pH 5.0/MeOH 80/20 (vol/vol) (phase A), and 50 mmol/L acetate buffer pH 5.0/MeOH/acetonitrile 40/40/20 (vol/vol/vol) (phase B). Channel voltages were ch1 20mV, ch2 100mV, ch3 200mV, ch4 270mV, ch5 470mV, ch6 550mV, ch7 670mV and ch8 720V. The following elution gradient was used using a total flow 0.3 ml/min.

HPLC gradient:

| Time min | % B |
| --- | --- |
| 0 | 20 |
| 5 | 20 |
| 7 | 30 |
| 12 | 30 |
| 20 | 50 |
| 42 | 50 |
| 45 | 65 |
| 57 | 65 |
| 63 | 100 |
| 78 | 100 |
| 83 | 20 |
| 98 | 20 |

6-ME gave peaks on two channels (ch3 and ch7) at retention time of 58.5 minutes. The total run time was 98 minutes. After HPLC runs, to 50µl (=1/2) of the samples deuterated internal standard (d6-6-MeEquol) was added. The samples were evaporated to dryness, silylated with 100µl of silylation reagent (pyridine:hexamethydisilazane:trimethychlorsilane 9:3:1 vol:vol:vol) for 30 min at room temperature and 1µl was injected in GC/MS.

**Effect of 6-ME on primary human fibroblast proliferation.** A. Human fibroblasts were split into 12-well plates at 5,000 cells per well and 24 h later cells were treated or not with various concentrations of 6-methoxyequol. After 2 days, cells were again treated or not with 6-ME and the next day cells were counted. B. Human fibroblasts were grown on coverslips and treated with 6-ME (10 and 20μΜ) for 24 h. BrdU was added 6 h before the 6-ME treatment was complete. Cells were fixed in 3.7% paraformaldehyde, quenched with 50mM ammonium chloride for 15 min, permeabilized with 0.1% Triton X-100 for 4 min, and non-specific sites were blocked with fetal serum. The proliferating cells were detected with an anti-BrdU antibody. Coverslips were mounted in Mowiol and viewed using Leica DM IBRE microscope.


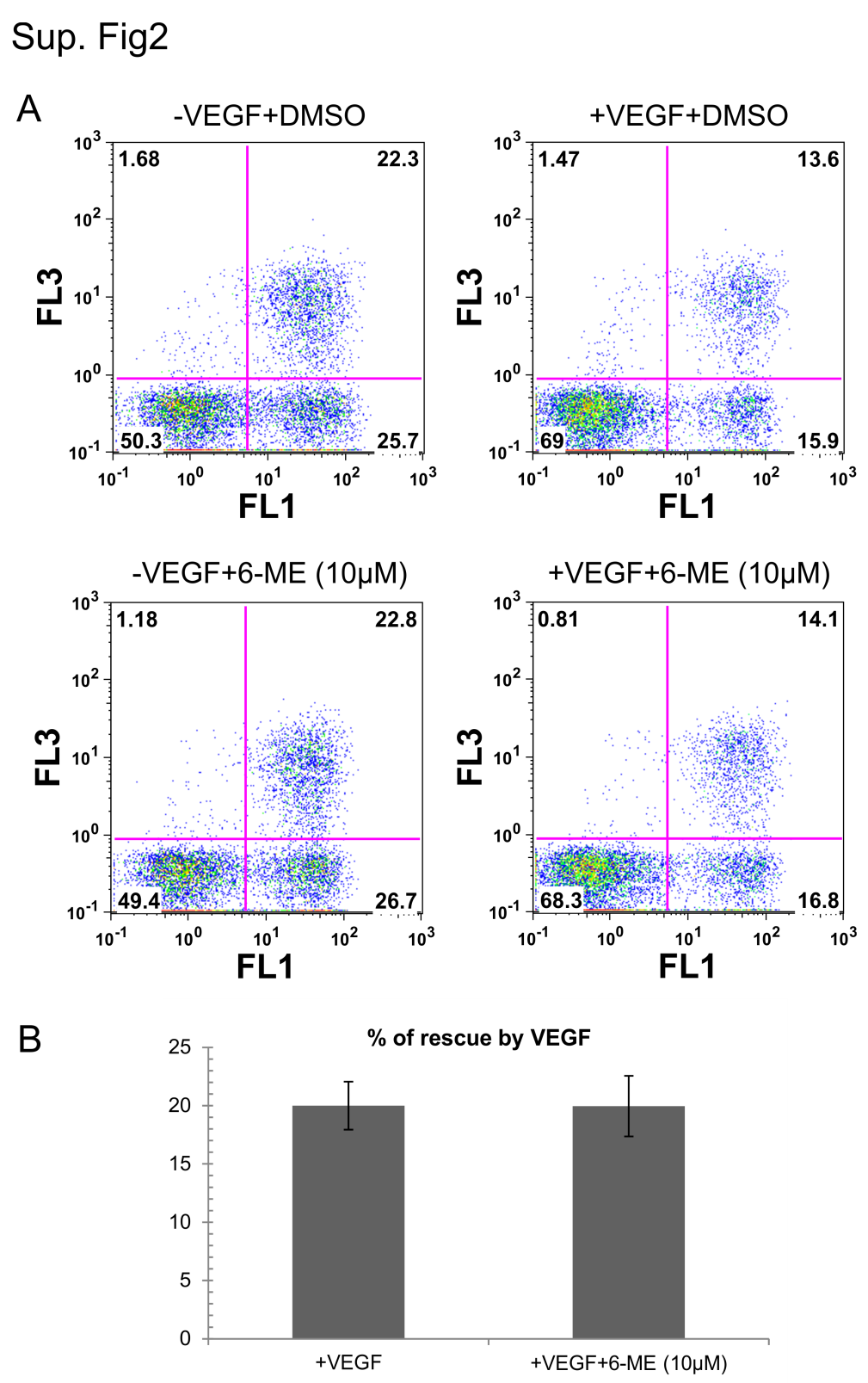


**Effect of 6-ME on VEGF-induced survival of endothelial cells**. A. HUVECs were serum starved for 6h in M199 supplemented with 5% FCS and were treated with VEGF (50 ng/ml) in the absence or presence of 6-ME (10μΜ) for 18 h. At the end of the incubation time, floating and adherent cells were collected and analyzed by flow cytometry using Annexin V-FITC (1.0 μg/ml) binding and PI (4.0 μg/ml) staining for 20 minutes.


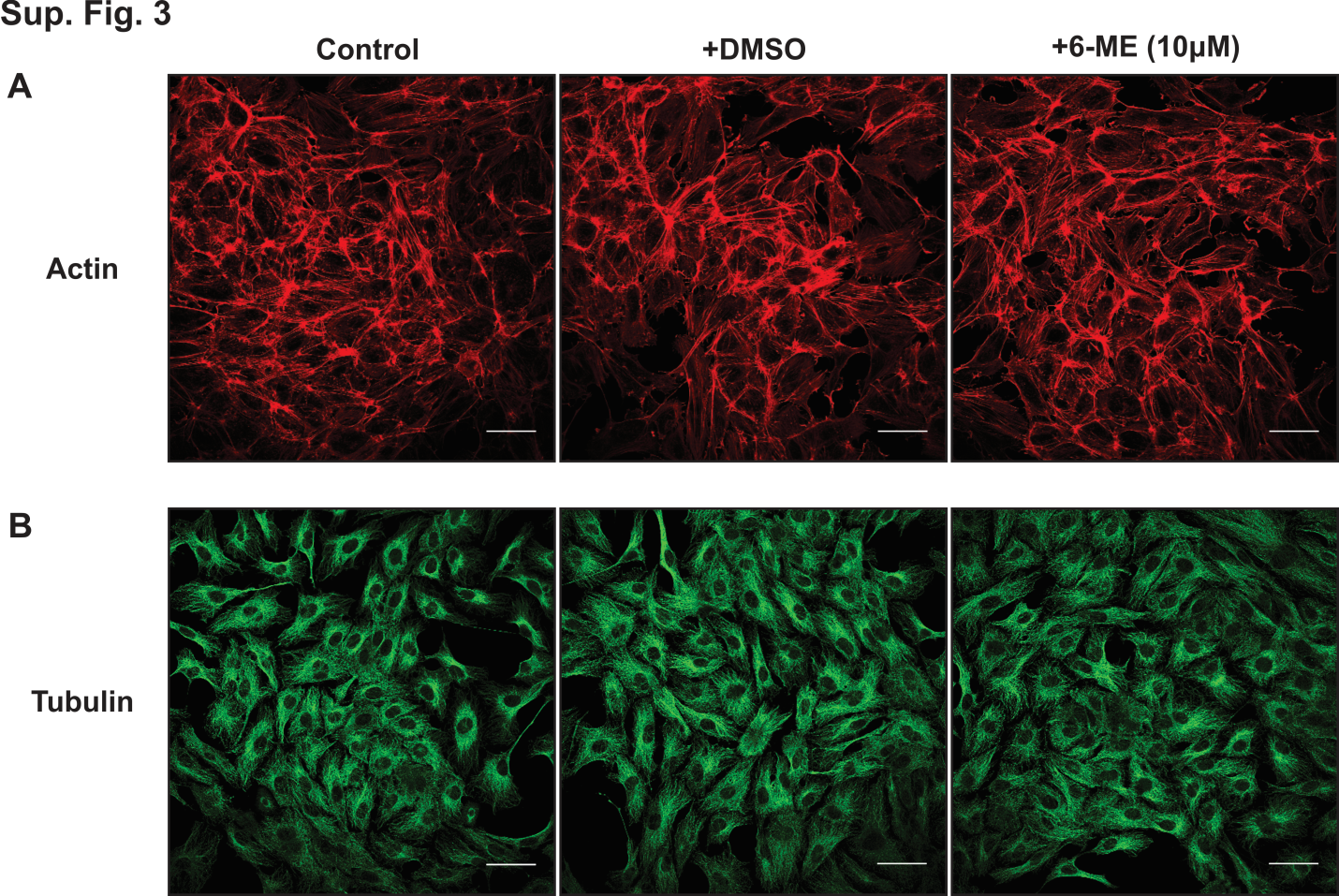


**Effect of 6-ME on the structure of actin filaments and microtubules.** HUVECs were treated with 6-ME (10μΜ) for 24 h before fixation with paraformaldehyde. Then, the samples were either stained with FITC-phalloidin (A) or used for indirect immunofluorescence with primary antibody that recognizes the endogenous tubulin (B). Cells were viewed with a Leica TCS-SP5 scanning laser confocal microscope equipped with an Argon laser (for excitation at 488), solid state 561 laser line and Helium Neon laser (for excitation at 633). Objective used was Leica 63 x HCX PL APO 1.3 NA. Images were acquired using Leica software and files were subsequently compiled in ImageJ and Adobe Photoshop


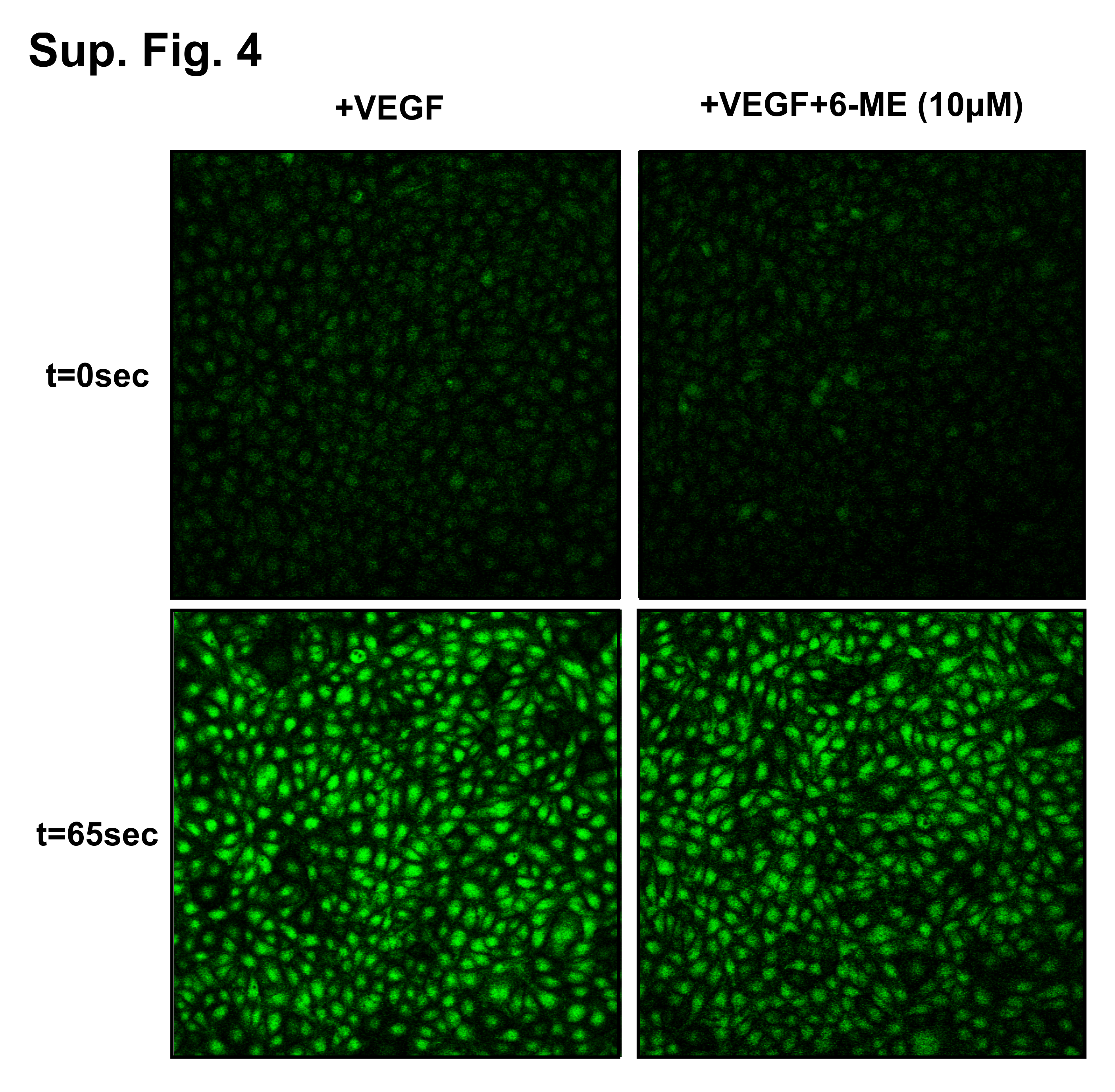


**Effect of 6-ME on VEGF-induced Ca^2+^ release.** HUVECs were grown in full medium until they reached 80% confluence. After removing culture medium, cells were incubated for 30 min at 37°C in a HBSS buffer containing Fluo-4 NW, 20mM HEPES, 2.5mM probenecid, and 1.5mM CaCl_2_ for. The plates were then placed into a pre-warmed cell culture chamber under a Leica SP5 confocal microscope and fluorescence was measured at the indicated times after VEGF (50ng/ml) administration using excitation and emission filers at 485 and 520 nm, respectively.
